# Geometrical restoration during total hip arthroplasty is related to change in gait pattern - a study based on computed tomography and three-dimensional gait analysis

**DOI:** 10.1186/s12891-021-04226-4

**Published:** 2021-04-20

**Authors:** A-C Esbjörnsson, S. Kiernan, L. Mattsson, G. Flivik

**Affiliations:** 1 Department of Clinical Sciences Lund, Orthopaedics, Lund University, Skane University Hospital, 221 85 Lund, Sweden; 2 Prophysics SOL, Jungmansvägen 3, 24335 Höör, Sweden

**Keywords:** Hip osteoarthritis, Total hip arthroplasty, Gait analysis, Computed tomography, Femoral neck anteversion, Femoral offset, Hip rotation, Hip joint moments

## Abstract

**Background:**

The effect of change in hip anatomy on change in gait pattern is not well described in current literature. Therefore, our primary aim was to describe and quantify changes in hip geometry and gait pattern 1 year after total hip arthroplasty (THA) in individuals with hip osteoarthritis. Our secondary aim was to explore the effect of postoperative change in femoral neck anteversion (FNA) and femoral offset and acetabular offset (FO/AO) quota on postoperative change in hip rotation and hip adduction moment during gait, respectively, 1 year after THA”.

**Methods:**

Sixty-five individuals with primary hip osteoarthritis, scheduled for THA, were analyzed in this prospective intervention study. Participants were evaluated pre- and 1 year postoperatively with computed tomography-scans, three-dimensional gait analysis, and patient-reported outcome measures. Multiple linear regressions were performed to evaluate the association between change in joint anatomy and change in gait pattern after THA.

**Results:**

One year postoperatively, global offset was symmetrical between sides as a result of decreased acetabular offset and increased femoral offset on the operated side. Quality of overall gait pattern improved, and participants walked faster and with less trunk lean over the affected side. FNA and hip rotations during walking changed equally in external and internal directions after THA and change in hip rotation during walking was associated with change in FNA in the same direction. An increase in external hip adduction moments was, on the other hand, not associated with change in FO/AO quota but with a more upright walking position and increased walking speed.

**Conclusions:**

The findings of this study suggest that geometrical restoration during THA impacts postoperative gait pattern and, in addition to known factors such as FO, height of hip rotation center, and leg length discrepancy, the FNA must also be taken into consideration.

**Trial registration:**

Trial registration: Clinicaltrial.gov, NCT01512550, Registered 19 January 2012 - Retrospectively registered.

## Background

Osteoarthritis of the hip joint is a progressive joint disease often leading to pain, muscular weakness, decreased function, and in the longer perspective decreased quality of life [1–3]. Symptoms are often eliminated or significantly reduced by total hip arthroplasty (THA) in which the joint is replaced with a prosthetic stem and cup. Common gait deviations in individuals with hip osteoarthritis are reduced sagittal plane motion, hip adduction moments, and hip joint rotation in the transverse plane [4–6]. Despite well-documented improvements following hip replacement surgery, long-term deviations in gait and function often persist, possibly due to muscular weakness [7] and compensatory movements [8–10].

**Fig. 1:**
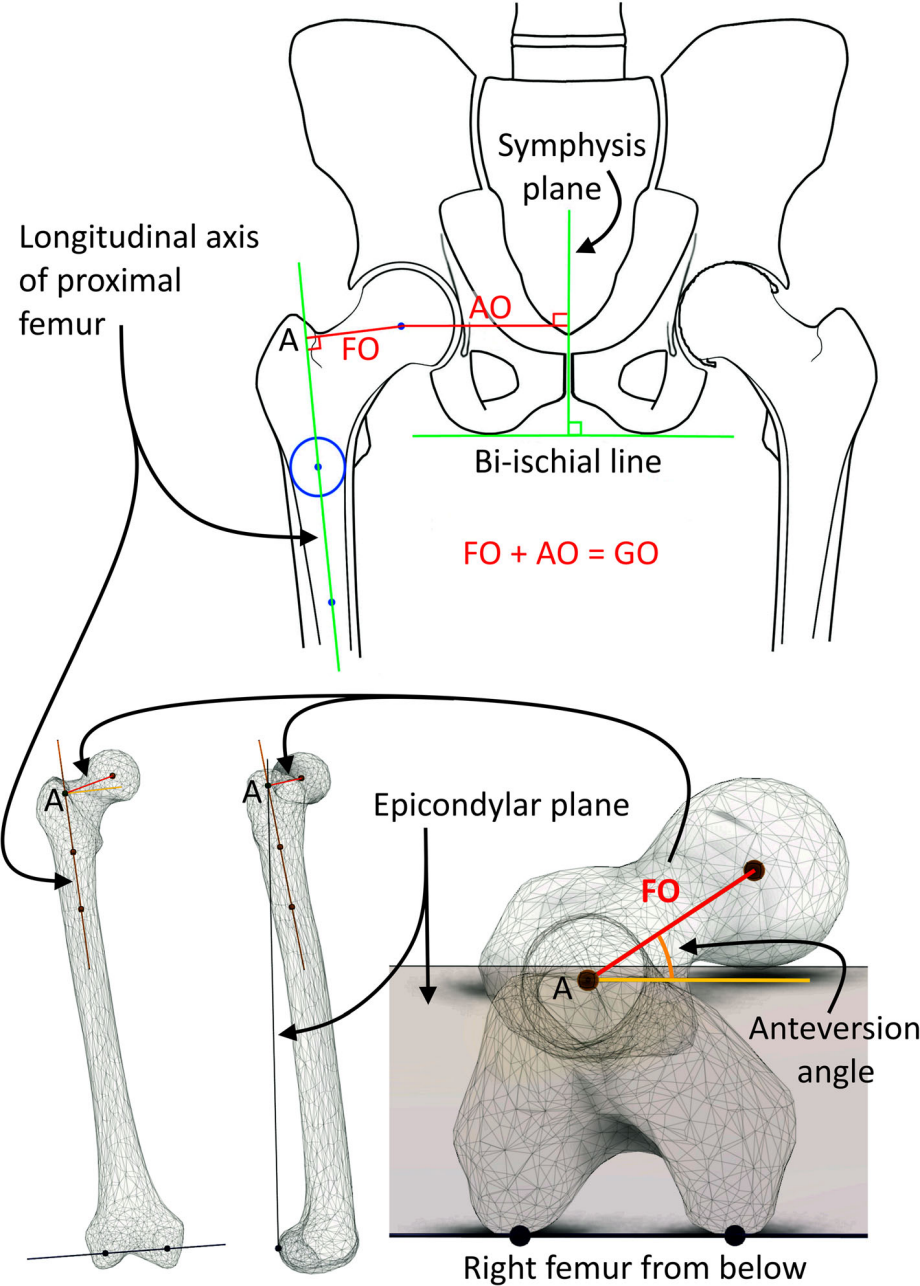
Definitions of CT measurements in the frontal and transversal planes. FO, Femoral Offset; AO, Acetabular Offset; GO, Global Offset; Anteversion, Femoral Neck Anteversion. Illustrations by Sverrir Kiernan

To optimize outcome, function of the hip must be restored during THA. In order to facilitate hip abductor strength, regain symmetry in global offset (GO) between sides and achieve soft-tissue balance around the hip joint, a change in the balance between acetabular offset (AO) and femoral offset (FO) is often required and advocated (Fig. 1) [11]. A decrease in AO is usually the result of reaming the acetabulum and medializing the cup, all within a safe zone defined for patients individually [12]. However, medialization of the cup reduces GO, and to restore the latter, a stem with offset greater than the natural offset of the femur is required. The compensatory increase of FO is considered important as this strategy appears to reduce polyethylene wear [13], improve prosthetic stability [14], and soft tissue tension [15]. Moreover, restoring FO has a positive effect on isometric hip abductor strength [16, 17], walking speed, and knee flexion and extension during walking 1 year after THA [18]. Restored FO has also been shown to influence knee joint moments but has no apparent impact on hip joint moments [19]. Most studies have focused on the FO in relation to gait and function. However, both the FO and AO are important to consider when restoring hip joint anatomy. In our study, we combined the FO and AO into a quota (FO/AO quota), to evaluate its effect on gait. Unlike FO, the AO/FO quota is a relative measure and thus independent of the size of the pelvis.

When orienting the stem in anteversion (FNA), the anteversion of the cup must also be taken into consideration, since the combined anteversion is of importance when considering the risk of hip impingement and dislocation [20]. According to previous studies, the FNA and cup anteversion safe zone is about 15–25° degrees, respectively. However, the estimates of the safe zone differ greatly in the literature depending on the varying geometrical definitions of the measurements as well as on the surgical approach and prosthetic types [21, 22]. To achieve the proposed safe zone, sometimes the FNA needs to be changed substantially from its original orientation during surgery, possibly shifting the hip rotation range of motion for the individual. Earlier studies show no association between change in FNA and self-rated function or pain [21, 23]. However, the association between changes in FNA and a person’s gait pattern, is not extensively described in current literature.

Our primary aim was to describe and quantify changes in hip geometry and gait pattern 1 year after THA in individuals with hip osteoarthritis. Our secondary aim was to explore the effect of postoperative change in FNA and FO/AO quota on postoperative change in hip rotation and hip adduction moment during gait, respectively, 1 year after THA”.

## Methods

### Study design and participants

A cohort of 75 individuals with unilateral primary hip osteoarthritis were recruited for this prospective intervention study. The study was part of a comprehensive study investigating different aspects of THA. The participants underwent THA between October 2009 and September 2011. Inclusion criteria were: < 75 years of age, primary unilateral osteoarthritis of the hip, ability to commit to the conditions of the study, including repeated CT and gait analysis evaluations. Exclusion criteria were: previous major orthopedic surgery in the lower limbs, other lower extremity joint pain or severe back pain, spinal deformities, rheumatoid arthritis, diabetes mellitus, neurologic disease, BMI > 40, and/or other conditions affecting walking ability. Participants were recruited from the THA waiting list at the department of orthopedics, Skåne University Hospital, Lund, Sweden. All enrolled participants provided written and verbal informed consent to participate in all parts of the study in accordance with the Declaration of Helsinki. The regional ethical review board at Lund University, Sweden, approved the study (Dnr:2009/369). Identifier number at ClinicalTrials.gov: NCT01512550.

Participants were evaluated with low dose CT scans, 3D gait analysis, and patient-reported outcome measures within 1 month prior to THA and again 1 year postoperatively. Reasons for failure to follow-up and/or exclusion were: periprosthetic fracture (*n* = 1), inability to walk independently because of other conditions (*n* = 2), no preoperative CT (*n* = 2), no postoperative gait analysis (*n* = 1) or CT (*n* = 2), and THA of contralateral hip within 6 months (*n* = 2). Thus, 65 participants remained for this study and were included in the statistical analysis.

### Surgical procedure and rehabilitation

Two experienced hip surgeons performed the operations through a posterolateral approach using a cementless cup and stem (ABG II and Trident (Stryker Orthopedics, Mahwah, New Jersey, USA)). Based on preoperative 2D templating and CT measurements, geometrical restoration of FNA and GO was attempted with the contralateral hip as reference. Preoperative planning was based on calibrated digital plain radiographs using Sectra IDS7 PACS Orthopaedic PackageTM (Sectra AB, Linköping, Sweden).

Postoperative regimens allowed full weight-bearing immediately following surgery. After surgery, participants were encouraged to use an appropriate walking aid for 1 to 2 months to facilitate normal gait pattern and avoid limping. Participants participated in rehabilitation according to standard practice at the hospital and, thereafter, in a primary care setting of the patient’s choice.

### Computed tomography

In the current study, CT was performed using a low-dose technique, with an effective dose close to that of plain radiography [24], showing that even with increased image noise, excellent results can be achieved. This study was part of a comprehensive study project investigating different aspects of THA where all the added information that CT gives was essential. The project had approval from both ethical review board and the local the local radiation committee. An independent observer made all measurements on the pre- and postoperative 3D-CT data blinded to previous measurements, preoperative 2D templating, and the participants’ management. The pre- and postoperative 3D-CT data were assessed for lever arms (i.e., AO and FO) and rotatory positions of the hip and stems (i.e., FNA) using a CT based 3D templating software, Ortoma® Hip plan, version 1.0.0.26 (Ortoma, Gothenburg, Sweden). The 3D-analysis software produced repeatable measurements for GO, FO, AO, and FNA with near-perfect both inter- and intra-observer agreements [25]. The analyzed variables were FNA, GO, and the FO/AO quota on both sides. The following definitions for CT measurements were used: The long axis of the proximal femur was defined by the center of two best-fit intramedullary spheres, one on the distal level of the trochanter minor and the other 6 cm further down in the femoral shaft. True FO was defined as the perpendicular distance from the long axis of the proximal femur to the rotational center of the femoral head. We will refer to the intersection of the FO onto the proximal long axis of the femur as point A. The condylar plane was defined by the posterior subchondral joint surface of the medial and lateral femoral condyles projected proximally to point A. The FNA was defined as the angle between the condylar plane and the line between point A and the rotational center of the head of the femur. The symphysial plane was defined as a plane in the middle of the symphysis and perpendicular to the bi-ischial line. The AO was defined as the distance from the symphyseal plane to the rotational center of the femoral head. The GO was defined as the sum of the FO and AO (Fig. 1).

### Three-dimensional gait analysis

Three-dimensional gait analysis was conducted at the motion analysis laboratory in Lund, Sweden using a six camera Vicon MX40+ system (Vicon Motion Systems Ltd., UK) set at a capture frequency of 100 hz and one OR6–5 force plate (Advanced Mechanical Technologies inc, USA). Segment position of the trunk and pelvis, joint rotations of the hip and foot, external joint moments and time/distance parameters were calculated using the Plug-In-Gait model (Vicon Motion Systems Ltd., UK) [26]. Data were extracted for analysis using proCalc software (Vicon Motion Systems Ltd., UK). The following parameters from the gait analysis were selected based on an a priori hypotheses of their association with changes in the transverse plane (hip anteversion) and in the frontal plane (femoral, acetabular or global offsets): mean trunk obliquity in stance (°), mean pelvic obliquity and rotation in stance (°), mean hip rotation in single stance (°), mean foot progression in single stance (°), mean hip adduction moment in stance (Nmm/kg), and maximal hip adduction moment between initial contact and midstance (1st peak) and between midstance and foot-off (2nd peak) (Nmm/kg). The following time and distance parameters were included: walking speed (m/s) and time in single stance (s). To evaluate overall gait quality in the lower extremity, the Gait Deviation Index (GDI) was calculated for the operated side. The GDI is based on kinematics from the pelvis and the hip in all three planes, the knee and ankle in the sagittal plane and foot progression in the transversal plane [27]. GDI scores are interpreted as follows: a value of 100 or higher indicates a normal gait pattern, while each 10-point decrement below 100 indicates one standard deviation (SD) from normal gait (e.g., a GDI score of 80 indicates 2 SD from normal gait). Participants walked barefoot on a 10 m walkway and were instructed to walk in a self-selected speed. Enough trial walks were allowed for the participants to reach their customary gait pattern. Following this, three strides containing kinematic and kinetic data from each side were collected and subsequently analyzed. Gait parameters showed excellent intra-subject repeatability between strides and the statistical analysis was based on the mean of discrete values and variables from the three strides.

### Patient-reported outcome measures

Pre- and 1 year postoperatively, all participants completed the hip disability and osteoarthritis outcome score (HOOS) [28] and the EuroQol- Five Dimensions EQ-5D [29]. HOOS is a joint-specific self-assessment questionnaire; reliable for assessing baseline function and change over time in individuals with hip osteoarthritis. The questionnaire is divided into five subscales, and each subscale generates a score ranging from 0 to 100, where 0 represents “worst” and 100 “best” [28]. In this study, the subscale for pain was used. EQ-5D is a generic, reliable questionnaire used to evaluate health-related quality of life. In this study, the patient-rated health VAS scale from EQ-5D, ranging from worst health 0 to perfect health 100, was used in the analysis [29].

### Radiographic severity of hip osteoarthritis

Preoperative radiographs were collected according to standard procedures. Images were classified according to the modified Kellgren Lawrence grade ranging from 0 to 4, where 0 represents no osteoarthritis and 4 severe osteoarthritis [30].

### Statistical analysis

Statistical analyses were performed using the Statistical Package for Social Science, version 22 (SPSS Inc., Chicago, IL; USA). Demographics and disease characteristics were described using means and standard deviations (SD) or median and range or inter quartile range (IQR). Assumptions of data normality were verified using the Shapiro-Wilks test and Q-Q plots. A *p*-value below 0.05 was considered statistically significant.

To evaluate the differences between pre- and postoperative hip joint anatomy (CT measured) and variables derived from 3D gait analysis, a paired sample t-test was used. To evaluate differences between postoperative CT measures and reference values from the contralateral side, an independent t-test was used. The Wilcoxon’s signed-rank test was used for identifying pre and postoperative differences in HOOS pain and EQ. 5D VAS score.

Multiple linear regressions were performed to evaluate the relations between changes in joint anatomy (THA) and changes in gait pattern. Assumptions of linear relationship and multivariate normality were checked by scatterplots and by comparing the residuals vs. predicted values (i.e., the residuals had to be normally distributed around zero). In regression model 1, change in mean hip rotation in single stance was used as the dependent variable. Change in femoral neck anteversion, pelvic rotation, and walking speed between pre and post evaluations were included as independent variables. In regression model 2, change in max external hip adduction moment in the first 50% of stance was used as the dependent variable. Change in FO/AO quota, trunk obliquity, pelvic obliquity, and in walking speed between pre and post evaluations were included as independent variables. All variables were entered at the same time. Pain, subscale in HOOS, was initially included as an independent variable in both models but was excluded based on low response frequency (*n* = 55). However, pain was *not* a statistically significant variable in any model, and the results of the analyses were equivalent with pain excluded.

## Results

Demographic data are presented in Table 1. One year after surgery, the participants experienced less pain and increased health-related quality of life (Table 1). Apart from the periprosthetic fracture (excluded), no other major complications, such as deep infection, nerve injuries or dislocations, were observed during the first year of follow-up.

**Table 1:** Demographic characteristics and patient reported outcome measures

Total number (n)	65			
Gender male/female (n)	44/21			
Operated side right/left (n)	34/31			
Age at surgery (mean (SD)) (yrs)	58.9 (8.4)			
BMI at surgery (mean (SD)) (kg/m^2^)	27.7 (3.9)			
Kellgren Lawrence score (median (range))				
OP side	3 (1–4)			
Contralateral side	0 (0–2)			
	**Pre**	**Post**	**Δ pre-post**	***p*-value**
HOOS, subscale pain (median (IQR)), *n* = 55	44 (35–55)	95 (83–100)	43 (33–60)	**< 0.001**
EQ-5D, state of health (median (IQR)), *n* = 56	70 (50–85)	85 (75–95)	15 (2–30)	**< 0.001**

*n* number, *yrs* years, *IQR* inter quartile range, Δ median difference, *EQ-5D* Euro-Qol 5 dimensions

### Changes in hip joint anatomy 1 year after THA

**Fig. 2:**
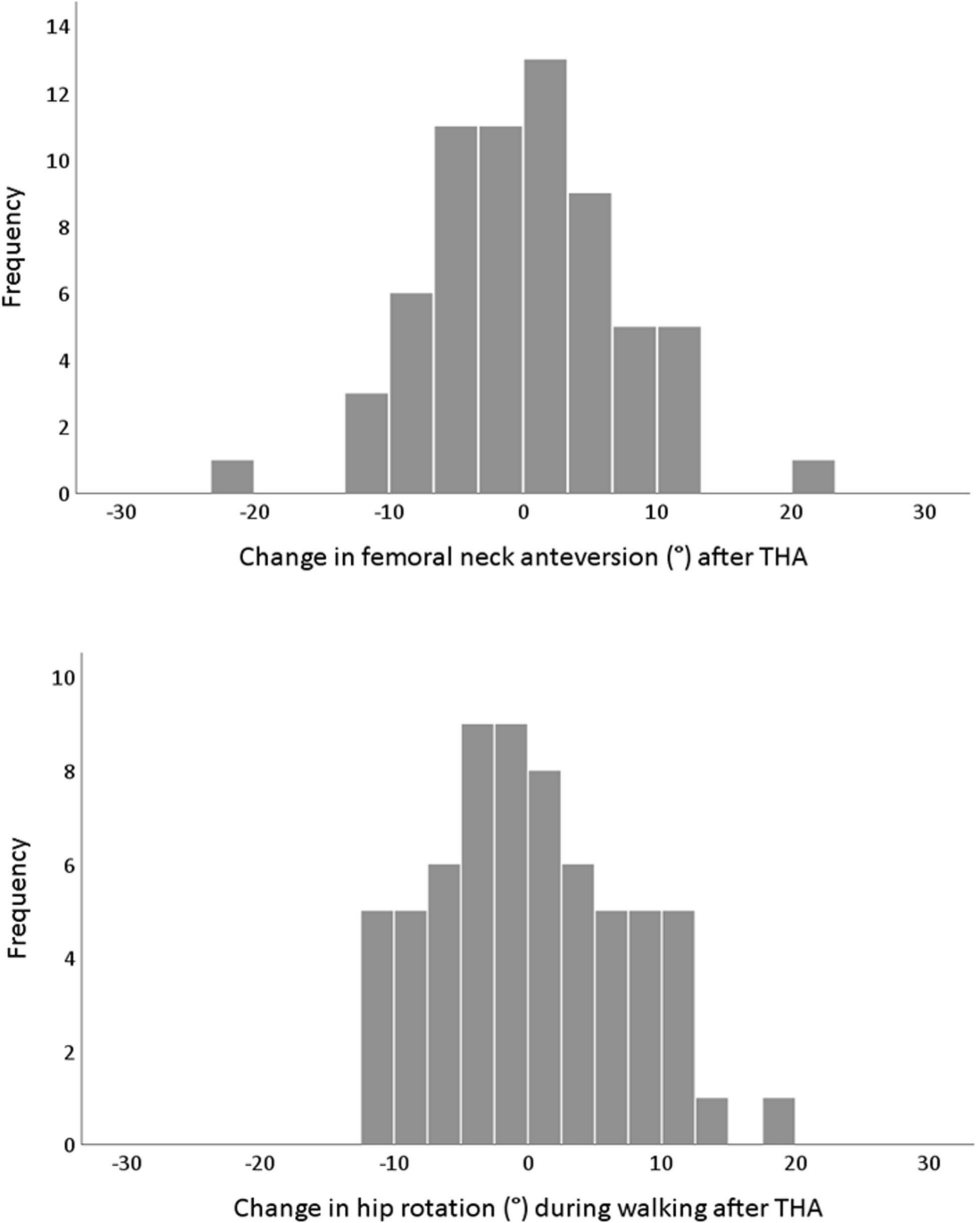
Histograms describing change in CT measured femoral neck anteversion and hip rotation during walking after THA. Positive values indicate internal rotation and negative values indicate external rotation

Compared to preoperative values, FNA angles changed equally in internal and external directions leading to a statistically non-significant change on group level (Fig. 2). Compared to preoperative values, the FO and FO/AO quota increased, while AO and GO decreased (Table 2). Postoperatively, compared to the contralateral side, FO had increased, and AO had decreased on the operated side while symmetry between sides were noted in FNA and GO (Table 2). The postoperative differences between sides in FO were distributed as follows: In 74% of the participants, the femoral offset of the operated side was within +/− 5 mm of the FO of the non-operated side. In 26%, the FO on the operated side was more than 5 mm longer than that of the non-operated side (increased FO > 5 mm). None of the participants had an FO which had decreased by more than 5 mm compared to the FO of the non-operated side (decreased FO > 5 mm).

**Table 2:** CT scan parameters, preoperative values for both sides (contralateral side for reference) and post-operative values for operated side

*n* = 65	Contralateral ref. values	Pre THA	Post THA	Mean diff. post vs pre mean [95% CI]	Mean diff. post vs. contralateral mean [95% CI]
Anteversion (°)	33.8 (10.2)	33.7 (10.0)	33.7 (9.6)	0.1 [−1.7, 1.9]	- 0.1 [−3.5, 3.4]
Acetabular offset (mm)	91.9 (5.0)	95.0 (5.2)	89.3 (4.3)	**−5.6 [−6.5, −4.8]**	**- 2.6 [−4.2, −0.9]**
Femoral offset (mm)	44.0 (6.2)	43.5 (6.6)	46.7 (6.2)	**3.2 [2.2, 4.2]**	**2.7 [0.6, 4.9]**
Global offset (mm)	135.9 (9.2)	138.5 (9.6)	136.1 (8.1)	**−2.4 [−3.4, −1.5]**	0.2 [− 2.8, 3.2]
FO/AO quota	0.48 (0.06)	0.46 (0.07)	0.52 (0.07)	**0.06 [0.05, 0.08]**	**0.04 [0.02, 0.07]**

Pre- and postoperative values are presented as mean (SD). Difference between pre- and postoperative values and between postoperative and contralateral values are presented as mean difference and 95% confidence interval. Statistically significant differences are highlighted in bold

*CT* computed tomography, *CI* confidence interval, *FO* femoral offset, *AO* acetabular offset

### Changes in gait pattern 1 year after THA

After THA, the quality of overall gait pattern, walking speed, and time spent in single stance increased significantly (Table 3). On the operated side, hip rotation during gait changed equally in internal and external directions leading to a statistically non-significant change on group level (Fig. 2). On the operated side, the pelvis segment became more externally rotated, and the foot segment became less externally rotated during stance. External hip adduction moments increased significantly, and participants walked with less trunk obliquity (i.e., less lean over the operated side). Pelvic obliquity decreased on the operated side, indicating less Trendelenburg gait, however this was not statistically significant (Table 3).

**Table 3:** 3D gait analysis parameters, pre- and postoperative data from the operated side

*n* = 65	Pre THA	Post THA	Mean diff. post vs. pre [95%CI]
**Overall gait pattern**			
Gait Deviation Index	81 (12)	90 (10)	**8.9 [5.7, 12.1]**
**Time and distance parameters**			
Walking speed (m/s)	1.02 (0.2)	1.14 (0.2)	**0.13 [0.1, 0.2]**
Time in single stance (s)	36.2 (3.3)	38.0 (1.7)	**1.8 [1.1, 2.5]**
**Gait variables hypothesized to be associated with femoral neck anteversion**			
Hip rotation (°)	0.3 (6.8)	0.3 (5.4)	0.0 [−1.7, 1.8]
Pelvic rotation (°)	0.8 (3.6)	−0.5 (2.5)	**−1.3 [−2.1, −0.4]**
Foot progression (°)	−10.0 (7.2)	−5.1 (6.1)	**4.9 [3.8, 6.1]**
**Gait variables hypothesized to be associated with femoral and acetabular offsets**			
Hip add mom avg. (Nmm/kg)	350 (88)	389 (88)	**40 [18.7, 60.5]**
Hip add mom peak 1 (Nmm/kg)	575 (134)	616 (122)	**40 [9.3, 71.6]**
Hip add mom peak 2 (Nmm/kg)	543 (120)	600 (133)	**57 [27.6, 85.5]**
Trunk obliquity (°)	−3.9 (2.4)	−3.0 (2.3)	**0.9 [0.3, 1.5]**
Pelvic obliquity (°)	2.7 (2.7)	2.1 (2.2)	−0.6 [−1.3, 0.1]
Hip adduction (°)	0.2 (3.1)	1.4 (3.1)	**1.2 [0.2, 2.1]**

Pre- and postoperative values are presented as mean (SD). Difference between post- and preoperative values are presented as mean difference and 95% confidence interval. Statistically significant differences are highlighted in bold

*THA* total hip arthroplasty, *CI* confidence interval, *add* adduction, *mom* moment, *avg.* average

### Relationship between change in hip anatomy and gait pattern

The change in hip rotation during gait after THA was associated with change in FNA, in the same direction, and with pelvic rotation, in the opposite direction, but not with change in walking speed (Table 4).

**Table 4:** Multiple linear regression analyses evaluating effect of change in geometrical restoration on change in gait pattern

Model 1. Multiple linear regression analysis, change in hip rotation during walking after THA defined as the dependent variable.				
*n* = 65	**Unstandardized B**	***p*-value**	**95% CI**	**R**^**2**^ **model**
**Change in hip anteversion**	0.34	**0.003**	**[0.12, 0.57]**	0.240
**Change in pelvic rotation**	−0.69	**0.004**	**[−1.15, − 0.23]**	
**Change in walking speed**	0.001	0.758	[−0.01, 0.01]	
Model 2. Multiple linear regression analysis, change in max hip adduction moment (1st peak) during walking after THA defined as the dependent variable.				
*n* = 65	**Unstandardized B**	***p*-value**	**95% CI**	**R**^**2**^ **model**
**Change in FO/AO (quota)**	4.02	0.985	[− 416, 424]	0.435
**Change in trunk obliquity**	17.39	**0.001**	**[7.01, 27.78]**	
**Change in pelvic obliquity**	17.98	**< 0.001**	**[8.81, 27.13]**	
**Change in walking speed**	0.23	**0.002**	**[0.08, 0.37]**	

Statistically significant differences are highlighted in bold. The Unstandardized B represents the amount by which the dependent variable changes if an independent variable is changed by one unit keeping other independent variables constant

*n* number, *THA* total hip arthroplasty, *CI* confidence interval, *FO* femoral offset, *AO* acetabular offset

The increase in hip adduction moment during gait was not associated with change in FO/AO quota but with less trunk lean and pelvic obliquity and an increase in walking speed (Table 4).

## Discussion

This study quantified changes in hip anatomy and gait pattern 1 year after THA compared to preoperative values. In addition, the effect of change in FNA on change in hip rotation during gait and the effect of change in FO/AO quota on change in external hip adduction moments were explored. We found that postoperatively, the GO was adequately restored, based on an increased FO and a decreased AO. Postoperative improvements were seen in gait pattern, pain and health-related quality of life. In addition, a change in hip rotation during walking was associated with change in FNA in the same direction and with change in pelvic rotation during gait in the opposite direction. An increase in external hip adduction moments was not associated with change in FO/AO quota, but with a more upright walking position and faster walking speed.

In agreement with previous research, walking speed and gait pattern improved 1 year after THA, but some gait deviations persisted, shown in this study by the postoperative GDI score of 90 (preoperative GDI score 81) [6, 8, 31]. The GDI is a summary score of gait deviations compared to that of a healthy reference group, taking the pelvis and lower extremity kinematics into account. After surgery, the participants walked more upright with less trunk lean over the operated side, indicating an increased ability to load the affected hip, which is not reflected in the GDI value. The more upright gait found in this study could, at least in part, be an effect of improved strength due to geometrical restoration after THA.

The importance of sufficient strength of the hip abductor muscles following THA has been widely discussed and agreed upon, including the effect of surgical approaches, compensatory movements and geometrical restorations [7, 8, 11, 15, 17, 18, 32, 33]. In the participants in the present study, the osteoarthritis had caused a slight increase in GO due to successive lateral migration of the femoral head by osteoarthritic hypertrophic changes in the acetabulum. In order to meet the goal of postoperative symmetry in GO between sides, the acetabulum on the operated side was reamed to be medialized, and the FO was increased [34]. This resulted in a significant increase in the FO/AO quota, potentially improving the biomechanical prerequisites for the hip abductor muscles [11, 35]. In the present study, an increase in external hip adduction moments was seen, but no association was found with the increase in FO/AO quota. As earlier stated, the participants walked more upright and faster after THA, which seems to have a greater impact on the external hip adduction moments during gait than the changed FO/AO quota. However, none of the participant had an FO on the operated side that was more than 5 mm shorter than on the non-operated side, indicating that all individuals had a restored or increased FO making it difficult to assess the possible adverse effects of a short FO on hip moments. Our results are in line with those of van Drongelen et al. (2019). They evaluated 22 individuals pre and post THA with biplanar radiographic examinations and 3D gait analysis and found no correlation between FO and hip adduction moments [19].

On group level, neither the average FNA nor the hip rotation during walking changed after THA. As shown in the histograms (Fig. 2), both variables changed equally in internal and external directions. It has been suggested that approximately 15–25° is a “safe zone” for FNA. The higher FNA angles presented here are an effect of our alternative anteversion CT measurement technique (Fig. 1). In this study, it was shown that changing FNA, had an impact on hip joint rotation during walking in equivalent direction. This means that if the THA is placed in more anteversion, the patient is likely to experience an increase in internal hip rotation during walking. Estimating the exact relationship between the amount of change in FNA and the consequent change in hip rotation during walking would be of great value for surgical planning. However, although 3D gait analysis is considered the gold standard for measuring gait and CT, the gold standard method for measuring FNA, such a direct relationship is very difficult to establish. The ability of the gait analysis model to accurately define the hip rotation center is of particular concern, as is the lower reliability of transversal plane rotation kinematics compared to the sagittal and frontal plane kinematics [36, 37]. Whereas measurements performed using 3D CT have shown high reproducibility and high consistency for both intra- and interobserver agreements [38, 39]. It was also found that change in hip rotation during gait was related to change in pelvis rotation in the opposite direction. This relationship is not unexpected since rotations of the hip joint and pelvis segment are linked. As an example, internal rotation of the pelvis during stance is typically accompanied by external rotation of the hip in order for the individual to maintain a straight line of progression. Since the rotations of the hip are defined and reported according to their relation to the pelvis segment in the biomechanical model used, this results in a negative correlation between the hip and pelvic rotations. The understanding of the relationship between change in FNA and change hip rotation during gait is further complicated by compensatory movements, pain, and muscular weakness. Therefore, in order to estimate the exact relationship, further studies are needed.

This study has some limitations and strengths. Leg length discrepancy after THA has been discussed as a cause of gait deviations [40]. In this analysis, we have not included this factor since leg length was measured on CT scans at the pelvis level, not on the total leg. It was therefore not possible to estimate the effect of changed leg length. For research purposes, CT measured total leg length should be included in future studies.

Inclusion of the height of the hip rotation center might also be considered since a high center of rotation decreases the lever arm and increases the force of the abductor muscles needed to balance the pelvis during walking [41]. After surgery, the FO was restored or increased in all of the participants. Thus, the impact of a decreased FO or FO/AO quota on gait pattern cannot be determined. The strength of this study includes the large group of participants and the increased precision in measurement offered by CT scans and 3D gait analysis. To the best of our knowledge, no other studies have used the FO/AO quota to quantify the ratio between the two lever arms acting around the hip joint. We believe this ratio to be a useful measure of the balance between the lever arms, with the added benefit of being relative and comparable between individuals, regardless of pelvis size.

## Conclusion

One year after THA, the GO was adequately restored, due to increased FO and a decreased AO. Postoperative improvements were seen in gait pattern, pain and health-related quality of life. In addition, a change in hip rotation during walking was associated with change in FNA in the same direction and with change in pelvic rotation during gait in the opposite direction. An increase in external hip adduction moments was not associated with change FO/AO quota but with a more upright walking position and increased walking speed. The findings of this study suggest that geometrical restoration during THA does impact postoperative gait pattern and, in addition to known factors such as FO, height of the hip rotation center and leg length discrepancy, the FNA must also be taken into consideration.

## Data Availability

The datasets that are used and analyzed for the present study are available from the corresponding author on reasonable request.

## Abbreviations

THA: Total Hip Arthroplasty
GO: Global Offset
FO: Femoral Offset
AO: Acetabular Offset
FNA: Femoral Neck Anteversion
CT: Computerized Tomography
3D: Thee-dimensional
2D: Two- dimensional
GDI: Gait Deviation Index
HOOS: Hip Osteoarthritis Outcome Score
EQ-5D: Euro-Qol 5 Dimensions
VAS: Visual Analog Scale
BMI: Body Mass Index
SD: Standard Deviation

## References

[1] Abramson SB, Attur M (2009). Developments in the scientific understanding of osteoarthritis. Arthritis Res Ther.

[2] Glyn-Jones S, Palmer AJ, Agricola R, Price AJ, Vincent TL, Weinans H, Carr AJ (2015). Osteoarthritis. Osteoarthritis Lancet.

[3] Loureiro A, Mills PM, Barrett RS (2013). Muscle weakness in hip osteoarthritis: a systematic review. Arthritis Care Res.

[4] Leigh RJ, Osis ST, Ferber R (2016). Kinematic gait patterns and their relationship to pain in mild-to-moderate hip osteoarthritis. Clin Biomech (Bristol, Avon).

[5] Watelain E, Dujardin F, Babier F, Dubois D, Allard P (2001). Pelvic and lower limb compensatory actions of subjects in an early stage of hip osteoarthritis. Arch Phys Med Rehabil.

[6] Ewen AM, Stewart S, St Clair Gibson A, Kashyap SN, Caplan N (2012). Post-operative gait analysis in total hip replacement patients-a review of current literature and meta-analysis. Gait Posture.

[7] Kiyama T, Naito M, Shinoda T, Maeyama A (2010). Hip abductor strengths after total hip arthroplasty via the lateral and posterolateral approaches. J Arthroplast.

[8] Bahl JS, Nelson MJ, Taylor M, Solomon LB, Arnold JB, Thewlis D (2018). Biomechanical changes and recovery of gait function after total hip arthroplasty for osteoarthritis: a systematic review and meta-analysis. Osteoarthr Cartil.

[9] Esbjornsson AC, Naili JE (2020). Functional movement compensations persist in individuals with hip osteoarthritis performing the five times sit-to-stand test 1 year after total hip arthroplasty. J Orthop Surg Res.

[10] Stief F, Schmidt A, van Drongelen S, Lenarz K, Froemel D, Tarhan T, Lutz F, Meurer A (2018). Abnormal loading of the hip and knee joints in unilateral hip osteoarthritis persists two years after total hip replacement. J Orthop Res.

[11] Clement ND, Patrick-Patel RS, MacDonald D, Breusch SJ (2016). Total hip replacement: increasing femoral offset improves functional outcome. Arch Orthop Trauma Surg.

[12] Bhaskar D, Rajpura A, Board T (2017). Current concepts in Acetabular positioning in Total hip Arthroplasty. Indian J Orthop.

[13] De Fine M, Romagnoli M, Toscano A, Bondi A, Nanni M, Zaffagnini S (2017). Is there a role for femoral offset restoration during total hip arthroplasty? A systematic review. Orthop Traumatol Surg Res.

[14] Forde B, Engeln K, Bedair H, Bene N, Talmo C, Nandi S (2018). Restoring femoral offset is the most important technical factor in preventing total hip arthroplasty dislocation. J Orthop.

[15] Ogawa T, Takao M, Hamada H, Sakai T, Sugano N (2018). Soft tissue tension is four times lower in the unstable primary total hip arthroplasty. Int Orthop.

[16] Mahmood SS, Mukka SS, Crnalic S, Wretenberg P, Sayed-Noor AS (2016). Association between changes in global femoral offset after total hip arthroplasty and function, quality of life, and abductor muscle strength. A prospective cohort study of 222 patients. Acta Orthop.

[17] McGrory BJ, Morrey BF, Cahalan TD, An KN, Cabanela ME (1995). Effect of femoral offset on range of motion and abductor muscle strength after total hip arthroplasty. J Bone Joint Surg Br.

[18] Sariali E, Klouche S, Mouttet A, Pascal-Moussellard H (2014). The effect of femoral offset modification on gait after total hip arthroplasty. Acta Orthop.

[19] van Drongelen S, Kaldowski H, Tarhan T, Assi A, Meurer A, Stief F (2019). Are changes in radiological leg alignment and femoral parameters after total hip replacement responsible for joint loading during gait?. BMC Musculoskelet Disord.

[20] Malik A, Maheshwari A, Dorr LD (2007). Impingement with total hip replacement. J Bone Joint Surg Am.

[21] Weber M, Weber T, Woerner M, Craiovan B, Worlicek M, Winkler S, Grifka J, Renkawitz T (2015). The impact of standard combined anteversion definitions on gait and clinical outcome within one year after total hip arthroplasty. Int Orthop.

[22] Murray DW (1993). The definition and measurement of acetabular orientation. J Bone Joint Surg Br.

[23] Muller M, Abdel MP, Wassilew GI, Duda G, Perka C (2015). Do post-operative changes of neck-shaft angle and femoral component anteversion have an effect on clinical outcome following uncemented total hip arthroplasty?. Bone Joint J.

[24] Geijer M, Rundgren G, Weber L, Flivik G (2017). Effective dose in low-dose CT compared with radiography for templating of total hip arthroplasty. Acta Radiol.

[25] Geijer M, Kiernan S, Sundberg M, Flivik G (2020). Pre- and postoperative offset and femoral neck version measurements and validation using 3D computed tomography in total hip arthroplasty. Acta Radiol Open.

[26] Davis RB, Ounpuu S, Tyberski SD, Gage JR (1991). A gait analysis data collection and reduction technique. Hum Mov Sci.

[27] Schwartz MH, Rozumalski A (2008). The gait deviation index: a new comprehensive index of gait pathology. Gait Posture.

[28] Nilsdotter AK, Lohmander LS, Klassbo M, Roos EM (2003). Hip disability and osteoarthritis outcome score (HOOS)--validity and responsiveness in total hip replacement. BMC Musculoskelet Disord.

[29] Brooks RG, Jendteg S, Lindgren B, Persson U, Bjork S (1991). EuroQol: health-related quality of life measurement. Results of the Swedish questionnaire exercise. Health Policy.

[30] Kellgren JH, Lawrence JS (1957). Radiological assessment of osteo-arthrosis. Ann Rheum Dis.

[31] Naili JE, Hedstrom M, Brostrom EW (2019). Changes of and interrelationships between performance-based function and gait and patient-reported function 1 year after total hip arthroplasty. J Orthop Traumatol.

[32] Petis S, Howard J, Lanting B, Jones I, Birmingham T, Vasarhelyi E (2017). Comparing the anterior, posterior and lateral approach: gait analysis in total hip arthroplasty. Can J Surg J Can Chirurgie.

[33] Sato H, Maezawa K, Gomi M, Kajihara H, Hayashi A, Maruyama Y, Nozawa M, Kaneko K (2020). Effect of femoral offset and limb length discrepancy on hip joint muscle strength and gait trajectory after total hip arthroplasty. Gait Posture.

[34] Terrier A, Levrero Florencio F, Rudiger HA (2014). Benefit of cup medialization in total hip arthroplasty is associated with femoral anatomy. Clin Orthop Relat Res.

[35] Charles MN, Bourne RB, Davey JR, Greenwald AS, Morrey BF, Rorabeck CH (2005). Soft-tissue balancing of the hip: the role of femoral offset restoration. Instr Course Lect.

[36] McGinley JL, Baker R, Wolfe R, Morris ME (2009). The reliability of three-dimensional kinematic gait measurements: a systematic review. Gait Posture.

[37] Zugner R, Tranberg R, Lisovskaja V, Shareghi B, Karrholm J (2017). Validation of gait analysis with dynamic radiostereometric analysis (RSA) in patients operated with total hip arthroplasty. J Orthop Res.

[38] Mao C, Liang Y, Ding C, Guo L, Wang Y, Zeng Q, Wang G (2016). The consistency between measurements of the femoral neck anteversion angle in DDH on three-dimensional CT and MRI. Acta Radiol.

[39] Sariali E, Mouttet A, Pasquier G, Durante E (2009). Three-dimensional hip anatomy in osteoarthritis. Analysis of the femoral offset. J Arthroplast.

[40] Bolink S, Lenguerrand E, Brunton LR, Hinds N, Wylde V, Heyligers IC (2019). The association of leg length and offset reconstruction after total hip arthroplasty with clinical outcomes. Clin Biomech (Bristol, Avon).

[41] Traina F, De Fine M, Biondi F, Tassinari E, Galvani A, Toni A (2009). The influence of the Centre of rotation on implant survival using a modular stem hip prosthesis. Int Orthop.

